# Optimization of Extraction Process of Polysaccharides MAP-2 from Opuntia Milpa Alta by Response Surface Methodology and Evaluation of Its Potential as α-Glucosidase Inhibitor

**DOI:** 10.3390/foods11213530

**Published:** 2022-11-06

**Authors:** Yan Yang, Maohui Yang, Xin Zhou, Huaguo Chen

**Affiliations:** 1 Key Laboratory for Information System of Mountainous Areas and Protection of Ecological Environment, Guizhou Normal University, 116 Baoshan North Road, Guiyang 550001, China; 2 Guizhou Engineering Laboratory for Quality Control & Evaluation Technology of Medicine, Guizhou Normal University, 116 Baoshan North Road, Guiyang 550001, China

**Keywords:** Opuntia Milpa Alta, polysaccharides MAP-2, optimization of extraction process, α-glucosidase inhibitor

## Abstract

The α-glucosidase inhibitors play an important role in blood glucose control in patients with type 2 diabetes. At present, the development of new α-glucosidase inhibitors is an urgent clinical need. Our previous studies have found that the polysaccharide MAP-2 in the cactus Opuntia Milpa Alta has significantly better activity than acarbose (one of the most widely used first-line α-glucosidase inhibitors in clinical practice), but its optimal extraction process parameters and inhibition kinetic characteristics are not clear, and whether it has the potential to become a new α-glucosidase inhibitors is also unclear. In this study, based on previous research, we used the combination of single factor experiments and the response surface method (RSM) to identify the optimal extraction conditions for MAP-2 as follows: solid-liquid ratio 1:4, extraction temperature 90 °C, extraction time 1 h. Under these conditions, the extraction yield of MAP-2 was 3.47 ± 0.062%. When the concentration of MAP-2 was 16 mg/mL, the inhibition rate of α-glucosidase was 91.13 ± 0.62%. In addition, the results of inhibition kinetics showed that the inhibition rate of MAP-2 on α-glucosidase was the highest at pH 7.4 for 30 min, and showed a good dose-effect relationship, which was a reversible competitive inhibition. Meanwhile, we also compared the activities of MAP-2 and acarbose on the side effects of acarbose related enzymes. Compared with acarbose, MAP-2 not only had a better activation effect on lactase, but also inhibited the activity of hyaluronidase, and the activation and inhibition rate were positively correlated with the concentration. However, under the same conditions, the effect of acarbose on hyaluronidase was opposite to that of MAP-2. At low concentration, acarbose had a certain activation effect on lactase, but gradually attained an inhibitory effect with the increase in concentration. In contrast, MAP-2 not only activates lactase activity, improves diarrhea, abdominal distension, and abdominal pain, but also inhibits hyaluronidase activity, to solve the side effects of allergic reactions, suggesting that MAP-2 has the potential to become a novel and effective inhibitor of α-glucosidase with fewer side effects.

## 1. Introduction

Diabetes mellitus (DM) is a common chronic metabolic disease [1], which is very harmful to human health [2]. The α-glucosidase inhibitors are a class of hypoglycemic drugs for the treatment of type II diabetes [3]. By inhibiting the activity of digestive enzymes in the brush border of small intestinal mucosa, these agents can delay the absorption of glucose, control the rapid rise of postprandial blood glucose concentration, effectively reduce postprandial blood glucose, and reduce the incidence of diabetes complications [4]. At present, the α-glucosidase inhibitors widely used in clinical practice mainly include acarbose [5], voglibose [6] and miglitol [7]. These drugs have significant hypoglycemic effects [8], but there are also different degrees of side effects, such as abdominal pain, diarrhea [9], allergy [10], intestinal colic, and intestinal disorders [11]. Therefore, the development of new α-glucosidase inhibitors with guaranteed efficacy and low side effects has become an urgent need.

Natural foodborne materials with high safety and low side effects [12] are considered as good sources for screening α-glucosidase inhibitors [13]. In recent years, polysaccharides have attracted extensive attention, and some polysaccharides have shown significant inhibitory effects on α-glucosidase. Zhang et al. found that polysaccharides of mulberry, known as “Folk holy fruit”, had a strong inhibitory effect on α-glucosidase [14]. Guo et al. also reported that corn silk polysaccharide had a good inhibitory effect on α-glucosidase, and its hypoglycemic activity was obvious in rats [15]. In addition, some other polysaccharides, such as those from *Mallotus furetianus* [16], Guava [14], and *Cordyceps militaris* [17], all showed good inhibitory effect on α-glucosidase activity.

In our previous work [18], six polysaccharide components were obtained from Opuntia Milpa Alta by different extraction methods. Through in vitro activity screening, it was found that one of the polysaccharide components (MAP-2) had a significant inhibitory effect on α-glucosidase, and its activity was significantly better than that of acarbose. The monosaccharide composition analysis showed that MAP-2 contained glucuronic acid, glucose, xylose, arabinose, and galacturonic acid. We believe that MAP-2 has the potential to become a good α-glucosidase inhibitor, worthy of further study. However, the optimal preparation process parameters of MAP-2 are not clear, and its dose-effect relationship and inhibition kinetics characteristics of α-glucosidase inhibition are also unclear. In addition to its superior inhibitory effect on α-glucosidase, does MAP-2 eliminate or reduce the side effects of acarbose? The existence of these problems is very unfavorable for the evaluation of MAP-2 as a good α-glycosidase inhibitor.

Therefore, this study used a combination of single factor experiments and the response surface method (RSM) to optimize the extraction process of MAP-2 and explore the dose-response relationship and inhibitory kinetic characteristics of MAP-2 on α-glucosidase, as well as the effects of MAP-2 on side-effect related lactase and hyaluronidase activities. Through these studies, to evaluate whether MAP-2 can significantly reduce blood glucose without causing serious side effects, and to provide a scientific basis for its in-depth development and utilization.

## 2. Materials and Methods

### 2.1. Materials and Reagents

The cactus cultivar Opuntia Milpa Alta was purchased from Suqian, Jiangsu Province and identified by Professor Chen Huaguo from Guizhou Normal University. Lactase, o-nitrophenyl-β-D-galactoside, α-glucosidase, p-nitrophenyl-β-D-glucopyranoside, PBS, and acarbose were purchased from Shanghai Yuanye Biotechnology Co., Ltd. (Shanghai, China). Petroleum ether (60–90 °C) and 95% ethanol were obtained from Tianjin Zhiyuan Chemical Reagent Co., Ltd. (Tianjin, China). Hyaluronidase, sodium hyaluronate, and cetyltrimethylammonium bromide (CTAB) were obtained from Soleibao. Sodium acetate, potassium dihydrogen phosphate, dipotassium phosphate, magnesium sulfate, and ethylenediaminetetraacetic acid disodium salt were purchased from Zhiyuan Biotechnology Co., Ltd. (Tianjin, China). All other reagents were analytically pure.

### 2.2. Sample Pretreatment 

A total of 28.0 kg of fresh Opuntia Milpa Alta leaves was weighed, cleaned, peeled, and squeezed into a slurry. It was placed in a 5000 mL round-bottom flask, added with petroleum ether (60–90 °C) and refluxed in a 65 °C water bath for 2 h. The ratio of material to liquid was 1:2, repeated twice. After the filter residue was washed with petroleum ether again for 2–3 times, it was placed in a 70 °C water bath to remove excess petroleum ether to obtain sample I.

### 2.3. Single Factor Experiment

In the previous study [18], we obtained the basic extraction process of MAP-2, that is, the pretreated Opuntia Milpa Alta sample was extracted with an appropriate amount of cold water (18 °C) and the residue was extracted with hot water. The obtained extract was then subjected to removal of impurities to obtain MAP-2. In this study, the 12.0 kg sample I was weighed, and the optimal cold water extraction parameter was determined as the solid-liquid ratio 1:2 for 2 h by single factor experiments, and the residue obtained after filtration was named as sample A. Further single factor experiments were carried out with sample A as the raw material, to determine the yield of MAP-2 and the inhibition rate of α-glucosidase as evaluation indexes.

Briefly, the ratio of solid to liquid was 1:4, the extraction time was 2 h, and the extraction temperatures of 60, 70, 80, 90, and 100 °C were investigated. Next, the fixed extraction temperature was set at 90 °C, extraction time was 2 h, and the solid-liquid ratios of 1:1, 1:2, 1:4, 1:6, and 1:8 were compared. Then the extraction temperature was fixed at 90 °C, the ratio of material to liquid was set at 1:4, and the effects of different extraction times (0.5, 1.0, 1.5, 2.0 and 3.0 h) on the extraction effect were analyzed. Finally, the extraction temperature was set at 90 °C, and with the ratio of material to liquid set at 1:4 and an extraction time of 1 h, the effects of number of extraction times (1, 2, 3, 4 and 5) on the evaluation index were investigated. All experiments were repeated three times.

### 2.4. RSM Experimental Design

After the preliminary determination of the extraction variables by single factor test, the solid-liquid ratio (*X*_1_), extraction time (*X*_2_) and extraction temperature (*X*_3_) were selected as independent variables, and the extraction yield of MAP-2 and the activity of inhibiting α-glucosidase were taken as response values. The RSM test was designed by using Design-Expert V12.0 software. The relevant design factors and codes are shown in Table 1. Experiment data were fitted to a second-order polynomial model and regression coefficients were obtained as follows:

$$Y=a_{0}+\overset{3}{\underset{i=3}{\sum }}a_{i}X_{i}+\overset{3}{\underset{i=3}{\sum }}a_{ii}X_{i}{}^{2}+\overset{3}{\underset{i<j=1}{\sum }}a_{ii}X_{i}X_{j}$$

where *a*_0_, *a_i_*, *a_ii_* and *a_ij_* were the regression coefficients for intercept, linear, quadratic and interaction terms, respectively, and *X_i_* and *X_j_* were the independent variables. The fitness of the second-order model was expressed by the regression coefficient R^2^ and its statistical significance was determined by *F* test. A *t*-Test was used for evaluating regression significance.

### 2.5. Inhibition Kinetics Experiment of MAP-2

#### 2.5.1. Effect of Reaction pH on α-Glucosidase Inhibition

Samples containing 50 μL of different concentrations of MAP-2 sample solution (0.5, 1.0, 2.0, 4.0, 8.0, 16.0 mg/mL), 0.1 U/mL α-glucosidase and 0.5 mmol/L PNPG solution (prepared by 0.1 mol/L PBS with pH 6.0, 6.4, 6.8, 7.2, and 7.4, respectively) were added to 96-well plates, respectively. After shaking, the reaction was carried out at 37 °C for 30 min, and then 100 μL 0.2 mol/L Na_2_CO_3_ solution was added. After incubation at 37 °C for 10 min, the absorbance value was measured at 405 nm and the inhibition rate was calculated.

#### 2.5.2. Effect of Reaction Time on α-Glucosidase Inhibition

According to the above determination method, 50 μL of MAP-2 solution with different concentrations (0.5, 1.0, 2.0, 4.0, 8.0, 16.0 mg/mL), 25 μL of 0.1 U/mL α-glucosidase and 0.5 mmol/L PNPG solution (prepared by 0.1 mol/L pH 7.4 PBS) were added to the 96-well plate in turn, and the reaction time was set to 10, 15, 20, 25, 30 min, respectively. The reaction was carried out in a constant temperature and humidity incubator at 37 °C, and then 100 μL of 0.2 mol/L Na_2_CO_3_ solution was added and incubated at 37 °C for 10 min. The absorbance value was measured at 405 nm and the inhibition rate was calculated.

#### 2.5.3. Determination of the Reversibility of Enzyme Inhibition by MAP-2

According to the above α-glucosidase inhibition determination method, the mass concentration of MAP-2 was set as 0, 2.0, 4.0, 8.0 mg/mL, the substrate concentration was 1.5 mmol/L, the reaction temperature was 37 °C, and the reaction time was 30 min. The enzymatic reaction rate of different α-glucosidase concentrations (0.1, 0.2, 0.4, 0.8 U/mL) was analyzed (the reaction rate was the ratio of absorbance value to time). The relationship curve between α-glucosidase concentration and enzymatic reaction rate was drawn to determine the reversibility of inhibition [19].

#### 2.5.4. Confirmation of the Inhibition Type of MAP-2

Under the conditions of 37 °C, reaction time 30 min and α-glucosidase concentration 0.1 U/mL, the enzymatic reaction rates of the different concentrations of MAP-2 solution (2.0, 4.0 and 8.0 mg/mL) with different concentrations of PNPG (0.5, 1.0, 1.5, 2.0 mmol/L) were determined, respectively. The inhibition type was determined by Lineweaver-Burk double reciprocal plot with 1/[S] and 1/V as abscissa and ordinate, respectively [20].

### 2.6. Determination of Lactase Activity

In this study, according to the reaction principle of o-nitrophenyl-β-D-galactoside and lactase, the lactase activity was determined by the ONPG test method [21]. Briefly, 1 mL of MAP-2 sample, 1 mL of acarbose solution (0.5, 1, 2, 4, 8, 16 mg/mL) and 1 mL of lactase solution (0.1 U/mL) were added to a 10 mL test tube, and balanced in a water bath at 30 °C for 10 min. Then 5 mL of ONPG (2.5 mg/mL) solution was quickly added, shaken and mixed. After 10 min of the water bath, 2 mL of 0.05 g/mL Na_2_CO_3_ solution was quickly added and mixed evenly. The absorbance value was measured at 420 nm. The lactase activity was calculated according to the following formula. At the same time, distilled water instead of the sample, was used as a blank control, and the same operation was used to determine the absorbance value.

$$Lactase\;activation\;\left(inhibition\right)rate\left(\%\right)=\frac{A_{1}-A_{2}}{A_{3}-A_{4}}\times 100{\%}_{\;}$$

where *A*_1_ is Absorbance of sample and lactate dehydrogenase solution, *A*_2_ is Absorbance of sample and reaction buffer, *A*_3_ is Absorbance of distilled water and lactate dehydrogenase solution, and *A*_4_ is Absorbance of distilled water and reaction buffer.

### 2.7. Determination of Hyaluronidase Activity

The anti-allergic effect of the MAP-2 sample was evaluated by the inhibition of hyaluronidase activity, and the test was conducted according to the method of Moller, et al. [22] with slight modification. Totals of 100 μL at different concentrations (0.5, 1.0, 2.0, 4.0, 8.0, 16.0 mg/mL) of the MAP-2 sample solution and 100 μL 1 mg/mL hyaluronidase (prepared by acetic acid-sodium acetate buffer) were added to 10 mL test tube in turn. After mixing, acetic acid-sodium acetate buffer (pH6.0, 0.2 mol/L) was added to 0.9 mL, and then water bathed at 37 °C for 15 min, then 100 μL 3.44 mg/L sodium hyaluronate solution was added, and the reaction was continued for 10 min. The reaction was terminated by adding 4 mL of 2.5% CTAB (dissolved in 2% NaOH solution), standing at room temperature for 15 min, then the absorbance value was measured at 405 nm, and the inhibition rate was calculated according to the following formula. Meanwhile, distilled water was used instead of the sample as blank control, and the absorbance value was determined by the same operation.

$$Inhibition\;rate\;of\;hyluronidase\left(\%\right)=\frac{A_{2}-A_{1}}{A_{4}-A_{3}}\times 100{\%}_{\;}$$

where *A*_1_ is the Absorbance of MAP-2 and hyaluronidase, *A*_2_ is the Absorbance of MAP-2 solution without hyaluronidase, *A*_3_ is the absorbance of distilled water and hyaluronidase, *A*_4_ is for distilled water and buffer absorbance.

### 2.8. Statistical Analyses

The results were expressed as mean ± standard deviation of three repeated measurements, and the extraction process parameters were optimized by Design-Expert 12.0 software. In vitro activity data were analyzed by one-way analysis of variance (ANOVA), and significant difference analysis was performed using IBM SPSS Statistics 20 and GraphPad Prism 8.0 software.

## 3. Results and Discussion

### 3.1. Single Factor Experiment

**Figure 1:**
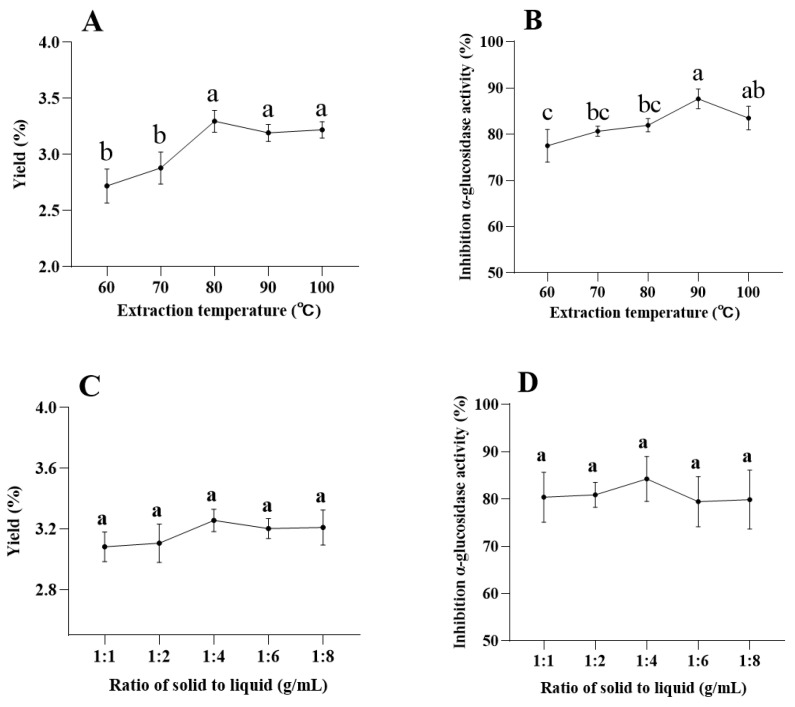

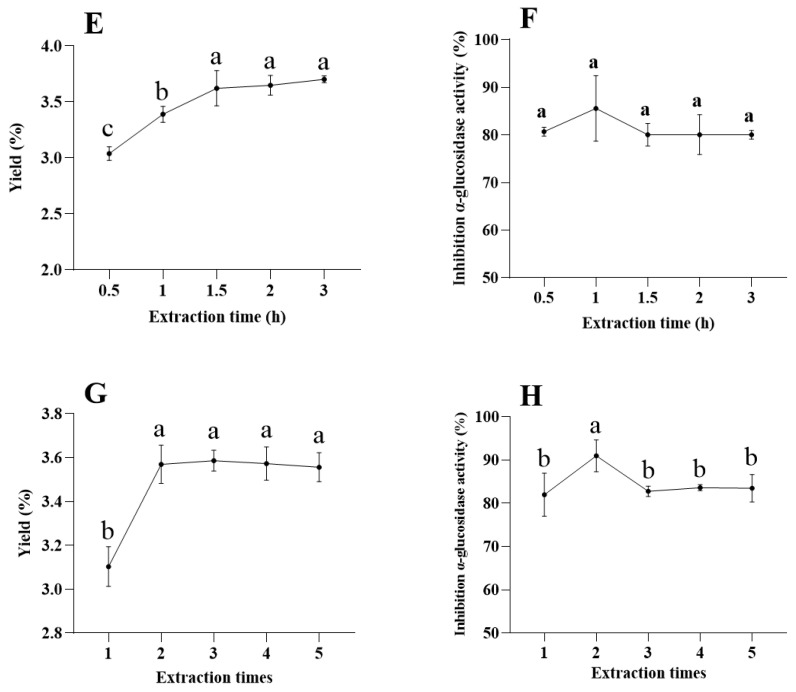
Effects of different extraction temperatures (**A**,**B**), solid-liquid ratios (**C**,**D**), time (**E**,**F**) and number of extraction times (**G**,**H**) on the yield of MAP-2 and inhibition of α-glucosidase activity. Each experiment was repeated three times; the error bars are standard deviations; significant (*p* < 0.05) differences are shown by data bearing different letters (a–c); statistical significances were carried out by ANOVA and Duncan’s test.

To explore the effects of extraction temperature, time, solid-liquid ratio, and extraction times on the extraction yield of MAP-2, and the inhibition of α-glucosidase activity by MAP-2, we conducted single factor experiments on these four factors. First, the effect of extraction temperature was investigated under the condition of fixed extraction time (2 h) and the solid-liquid ratio (1:4), and the results are shown in Figure 1A,B. When the temperature increased from 60 °C to 80 °C, the yield of MAP-2 increased continuously, and the maximum yield was obtained at 80 °C. When the temperature increased from 80 °C to 100 °C, the yield of MAP-2 decreased slightly, but not significantly. However, with the increase in temperature, α-glucosidase activity was greatly affected, and there were significant differences between different temperatures. The maximum inhibition rate was observed at 90 °C, which was much higher than that at 80 °C. Therefore, in the current experiment, we believe that 90 °C is more suitable for the extraction temperature. Figure 1C,D shows the effect of the solid-liquid ratio under a fixed extraction temperature (90 °C) and extraction time (2 h). With the increase in the solid-liquid ratio, the yield of MAP-2 and its inhibitory effect on α-glucosidase reached were maximized at 1:4, with values of 3.26% and 84.23%, respectively. But with the change in the solid-liquid ratio, the yield of MAP-2 and the inhibition rate of α-glucosidase did not change significantly. Under the conditions of extraction temperature of 90 °C and a solid-liquid ratio of 1:4, the inhibitory effect of MAP-2 on α-glucosidase reached a maximum value at 1 h with the prolongation of time, but there was no significant difference between them. The yield of MAP-2 increased with the increase in extraction time, and the best yield was obtained at 1.5 h, and there was no significant difference after this time, as shown in Figure 1E,F. Therefore, an extraction time of 1 h is most appropriate. Finally, under the conditions of fixed extraction temperature (90 °C), liquid-solid ratio (1:4), and extraction time (1 h), the effects of the number of extraction times were compared, and the results were shown in Figure 1G,H. The yield of MAP-2 and the inhibition rate of α-glucosidase were the best when extracted twice; increasing the number of extraction times did not further increase yield or inhibition activity.

### 3.2. RSM Analysis

#### 3.2.1. Model Fitting and Statistical Analysis

To further optimize the extraction process of MAP-2, on the basis of the above single factor experimental results, the solid-liquid ratio (*X*_1_, 1:2–1:6), extraction time (*X*_2_, 0.5–1.5 h) and temperature (*X*_3_, 80–100 °C) were optimized by RSM. According to the Box-Behnken module of Design-Expert 12.0 software, 17 groups (Table 1) of experimental schemes were designed, and the corresponding yield of MAP-2 and inhibition rate of α-glucosidase were obtained. On this basis, quadratic polynomial fitting was performed to obtain the fitting equation of MAP-2 yield (*Y*_1_) and α-glucosidase inhibition rate (*Y*_2_).

*Y*_1_ = 3.4 − 0.0125*X*_1_ + 0.04*X*_2_ + 0.5675*X*_3_ − 0.0225*X*_1_*X*_2_ + 0.0025*X*_1_*X*_3_ − 0.0325*X*_2_*X*_3_ − 0.3113*X*_1_^2^ − 0.1263*X*_2_^2^ − 0.2362*X*_3_^2^.

*Y*_2_ = 91.01 + 0.2175*X*_1_ + 0.0613*X*_2_ + 4.26*X*_3_ + 1.3*X*_1_*X*_2_ − 0.77*X*_1_*X*_3_ + 0.6025*X*_2_*X*_3_ − 1.42*X*_1_^2^ − 0.7288*X*_2_^2^ − 5.06*X*_3_^2^.

Analysis of variance (ANOVA) was used to evaluate the statistical significance of the coefficients of each model (Table 2 and Table 3). The determination coefficient R^2^ of the model was above 0.9, indicating that the model fitting was satisfactory, and the experimental value was not much different from the expected value [23].

As shown in Table 2, R^2^ is 0.98, and the modified determination coefficient is 0.96, indicating that the model can explain most of the changes in MAP-2 yield. In addition, the model had a higher F value (39.54) and a lower *p* value (<0.001), indicating that the model and related variables were extremely significant, and the *p* values of the three variables were *X*_3_ < *X*_2_ < *X*_1_. As shown in Table 3, the F of the model is 23.22, *p* < 0.001, and the model and related variables are extremely significant. Among the three variables studied, the *p* value was *X*_2_ > *X*_1_ > *X*_3_, indicating that the extraction temperature had the greatest influence on the yield of MAP-2 and the activity of α-glucosidase.

#### 3.2.2. Analysis of the Influence of Different Factors

In the RSM test analysis, the three-dimensional (3D) response surface and the two-dimensional (2D) contour map can be used to characterize the regression function between different influencing factors [24], and then show the type of interaction between the two test variables and the relationship between the response of each variable and the experimental level [25]. Among them, the contour map is circular or elliptical to illustrate whether the interaction between variables is significant. A circular contour map indicates that the interaction between the corresponding variables can be ignored, while an elliptical contour indicates the opposite [26]. In this study, the 3D response surface and 2D contour map were used to evaluate the effects of extraction time, temperature, and material liquid on the yield of MAP-2 and the inhibition of α-glucosidase activity, and the interaction between the test factors was described.

**Figure 2:**
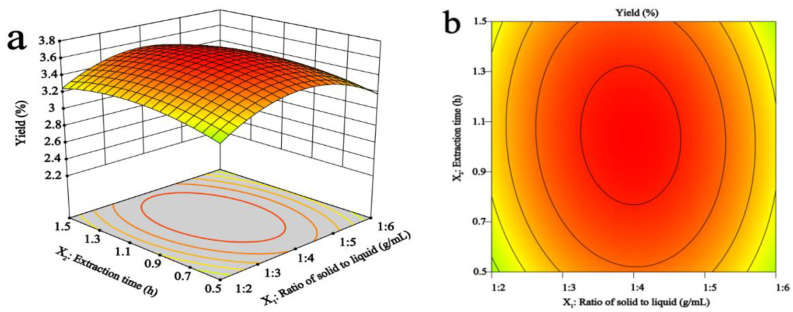

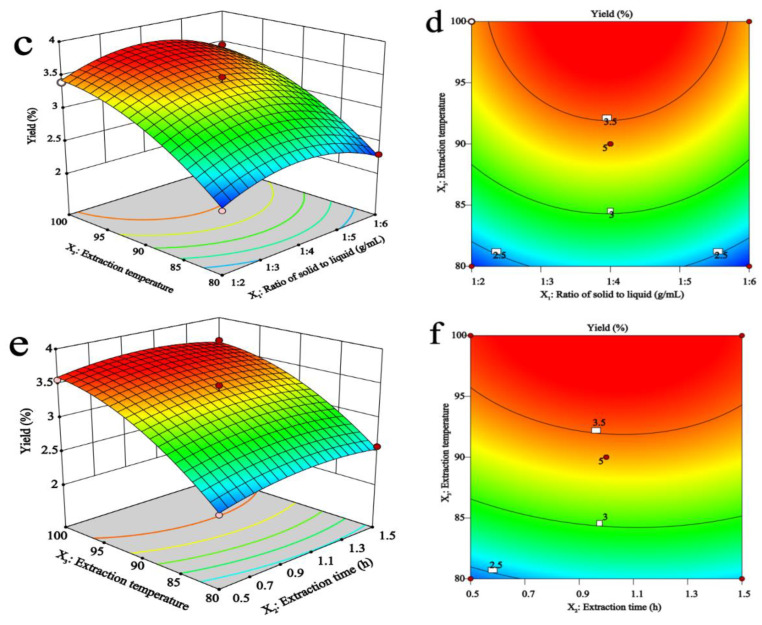
Effects of solid-liquid ratio, extraction time, and temperature on the yield of MAP-2. Effects of different extraction time and solid-liquid ratios (**a**,**b**), extraction temperatures and solid-liquid ratios (**c**,**d**), extraction temperatures and extraction time (**e**,**f**) on the yield of MAP-2.

Figure 2a,b shows the 3D response surface MAP and contour MAP drawn under different extraction time and the solid-liquid ratio at a fixed temperature. Within 0.5–1.5 h, the yield of MAP-2 did not change much, and the threshold level of the solid-liquid ratio of 1:4 reached the maximum value.

In Figure 2c,d, the extraction time was constant, and the 3D response surface and 2D contour maps of MAP-2 yield were drawn under different extraction temperatures and solid-liquid ratios. The yield increased rapidly with the increase in temperature, and the yield increased gradually with the increase in the solid-liquid ratio, but the yield did not continue to increase but decreased slightly after exceeding 1:5, which meant that the further increase in the solid-liquid ratio did not increase the yield of MAP-2.

Figure 2e,f shows the 3D response surface and 2D contour maps obtained according to different extraction time and temperature under the condition of fixed solid-liquid ratio. The yield of MAP-2 increases with the increase in temperature and time.

**Figure 3:**
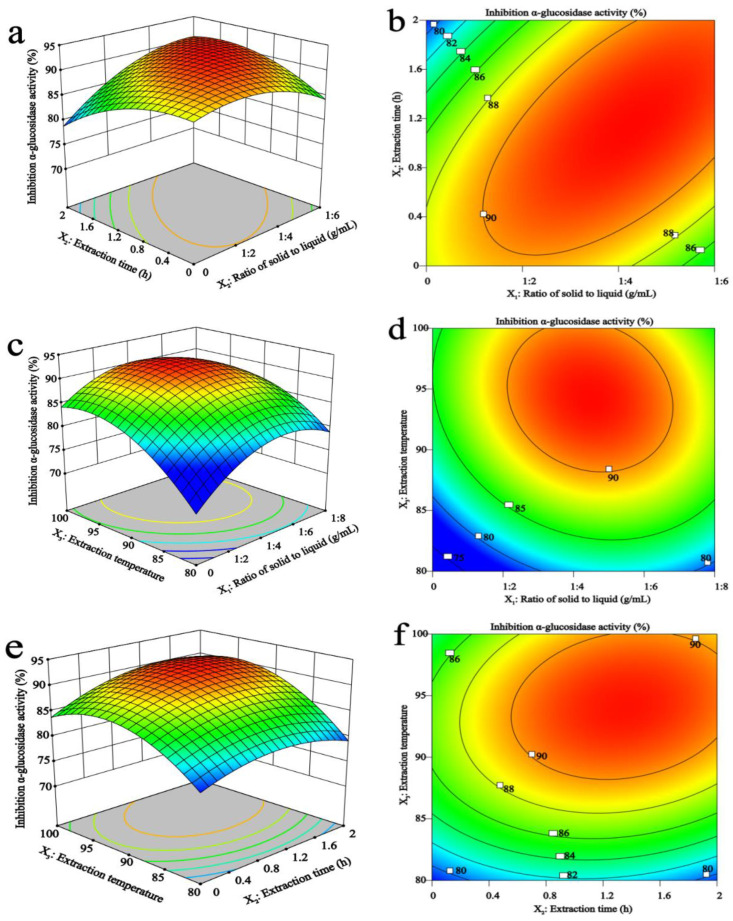
Effects of the solid-liquid ratio, time, and temperature on MAP-2 inhibition effect of α-glucosidase activity. Effects of different extraction time and solid-liquid ratios (**a**,**b**), extraction temperatures and solid-liquid ratios (**c**,**d**), extraction temperatures and extraction time (**e**,**f**) on MAP-2 inhibition effect of α-glucosidase activity.

However, in addition to considering the effect of different influencing factors on the yield of MAP-2, we also considered the effect of MAP-2 obtained under different influencing factors on the inhibitory activity of α-glucosidase, and the results are shown in Figure 3.

Figure 3a,b shows that when the temperature was constant, with the extension of extraction time, the inhibition rate reached the maximum at the threshold level of 1 h, and then decreased. With the increase in the solid-liquid ratio, the inhibition rate also increased, showing the most effective inhibition rate at 1:4.

Figure 3c,d well illustrates the interaction between extraction temperature and the liquid-solid ratio. When the solid-liquid ratio was fixed, the inhibition rate of MAP-2 on α-glucosidase increased rapidly with the increase in temperature. At the same time, the inhibition rate also increased with the change in the solid-liquid ratio. Finally, the ideal inhibition rate was obtained at 95 °C and 1:5.

Shown in Figure 3e,f, when the solid-liquid ratio was fixed, the 3D response surface map obtained was steeper with the increase in temperature and time, especially the extraction temperature, indicating that the temperature has a greater impact. When the temperature was 95 °C and 1.2 h, the inhibition rate reached the maximum.

#### 3.2.3. Verification of the Models

Using the selected optimal conditions, the adaptability of the model equation to predict the optimal response surface was verified. The maximum predicted and experimental benefit values for MAP-2 are shown in Table 4. Further experiments were carried out using the optimal extraction conditions of the predicted values: the extraction time was 1.1 h, the extraction temperature was 95 °C, and the solid-liquid ratio was 1:4. The maximum yield predicted by the model was 3.64%, and the maximum inhibition rate of α-glucosidase was 91.87%. In order to ensure that the predicted values were not biased towards the experimental values, the experimental review was performed using the best conditions of these modifications: the extraction time was 1 h, the extraction temperature was 90 °C, and the solid-liquid ratio was 1:4. The average yield obtained from the experiment was 3.47%, and the average inhibition rate was 91.13%, which verified the correctness of the RSM model.

### 3.3. Inhibition Kinetic Analysis

#### 3.3.1. Effect of Different pH and Time on α-Glucosidase Inhibition

**Figure 4:**
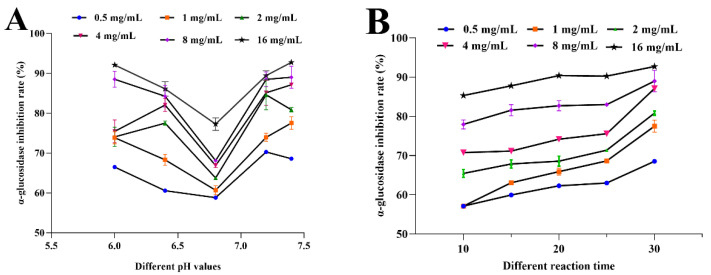
Effect of different pH and time on α-glucosidase activity. Effect of different pH values (**A**) and time (**B**) on α-glucosidase activity.

In our previous study, we found that under mild conditions, polysaccharide from Opuntia Milpa Alta had a good inhibitory effect on α-glucosidase in vitro. Therefore, this study further investigated the effects of a different extraction time and pH on α-glucosidase activity. As shown in Figure 4, the inhibition rate of MAP-2 on α-glucosidase was positively correlated with the concentration at the same reaction time and pH. As can be seen in Figure 4A, the inhibition rate of MAP-2 on α-glucosidase varied greatly at different pH levels in the range of polysaccharide concentration from 0.5–16 mg/mL. When pH was 6.8, the inhibition activity of MAP-2 on α-glucosidase was weak, while when pH was 6.0 and 7.4, the inhibition rate was high, which may be affected by the properties of polysaccharides [27]. As can be seen from Figure 4B, when the pH was fixed at 7.4, the inhibition rate of MAP-2 on α-glucosidase increased with the prolongation of reaction times. Before 25 min, the increase was relatively gentle, and after that, the increase was more obvious. However, overall, the inhibition rate of MAP-2 on α-glucosidase increased with the increase in concentration regardless of time or pH change. As was associated with *Ginkgo biloba* seed polysaccharides (15.54%) [28], pumpkin polysaccharide (17.8%) [29],and LH1 intracellular polysaccharides (iP) (62.00%) [30], MAP-2 had stronger α-glucosidase inhibitory activity.

#### 3.3.2. Inhibitory Kinetics Analysis of MAP-2 on α-Glucosidase

**Figure 5:**
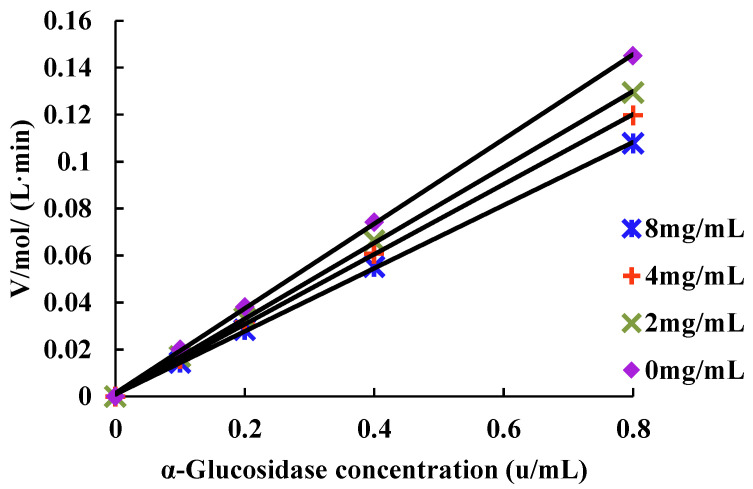
Linear fitting curve of the relationship between α-glucosidase and enzymatic reaction rate.

According to the combination of inhibitor and enzyme, the results can be divided into reversible inhibition and irreversible inhibition. Reversible inhibition is that the curve of rate and enzyme concentration passes through the origin [19], while irreversible inhibition is that the curve does not pass through the origin and has a positive intercept with the Y axis [31]. As shown in Figure 5, all the lines passed through the origin of coordinates, and the slope of the lines decreased successively as the mass concentration of MAP-2 increased. Therefore, the inhibitory effect of MAP-2 on α-glucosidase activity was reversible.

**Figure 6:**
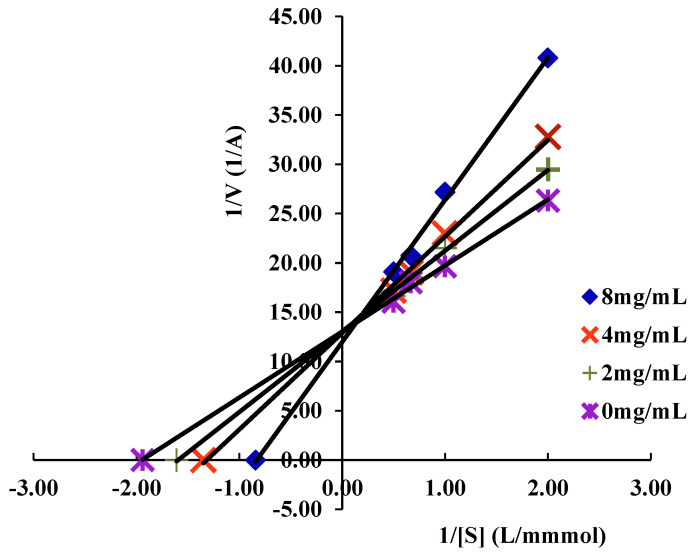
Lineweaver−Burk curve of MAP−2 inhibition of α−glucosidase activity.

To clarify the inhibition type of the MAP-2 inhibitor, an experimental model of PNPG with different concentrations of α-glucosidase was established in the presence of MAP-2. The Lineweaver–Burk double reciprocal plot is shown in Figure 6. With the increase in MAP-2 (2, 4, 8 mg/mL) mass concentration, the slope of the double reciprocal plot gradually increases, and the 1/[S] curve has good linearity. The double reciprocal fitting curve of the preparation is: (R^2^ = 0.9989), the Michaelis constant Km is 0.514, and the maximum rate of enzymatic reaction Vmax is 0.08. In addition, it can be seen from the Figure 6 that the inhibition curves of different horizontal intercepts and slopes intersect at one point with the longitudinal axis, indicating that the inhibitor concentration increases, Km increases, and Vmax remains unchanged, which is the most significant feature of competitive inhibition [32]. It was also shown that MAP-2 can bind to the active site of α-glucosidase in a reversible manner, thereby inhibiting the decomposition of glycosides into glucose and reducing postprandial blood glucose.

### 3.4. Determination of Lactase Activity

**Figure 7:**
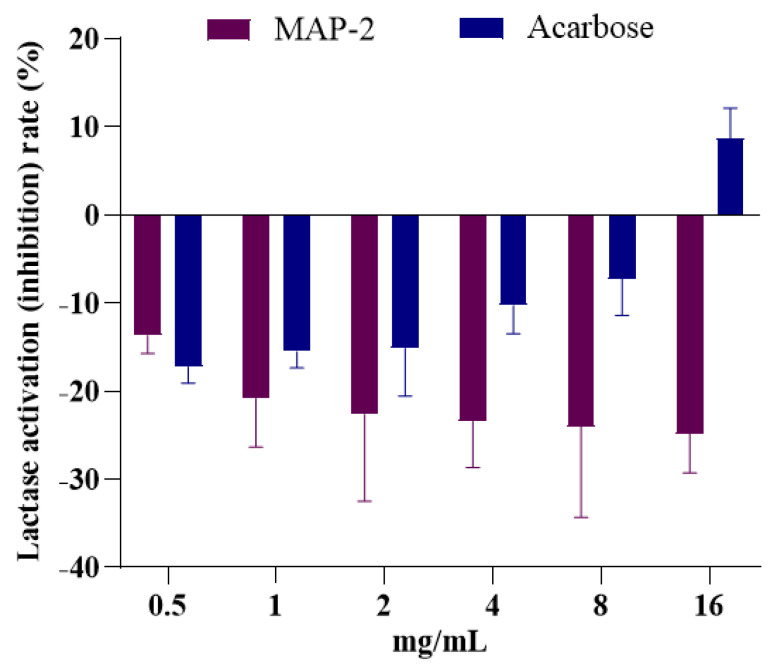
Effects of MAP−2 and Acarbose on lactase activity.

Lactase, also known as β-galactosidase, is one of the important functions of enzymes of the intestinal tract, mainly composed of small intestinal villus and intestinal bacteria secrete of brush border, and closely associated with diarrhea, abdominal distention [33], and studies have shown that, when the body lacks lactase, the symptoms of lactose intolerance occurs [34], such as severe diarrhea, abdominal distension [32], and severe cases can produce dehydration, acidosis and growth disorder, etc. [35]. As a commonly used inhibitor of α-glucosidase, acarbose has adverse reactions such as diarrhea, abdominal distension and abdominal distension [36]. Therefore, in this study, we established a lactase model to compare the effects of MAP-2 and α-glucosidase inhibitors on lactase activity in response to diarrhea, bloating, and abdominal distension in acarbose, as shown in Figure 7. In the range of 0.5–16 mg/mL, MAP-2 not only activated lactase, but also the activation rate increased with the increase in concentration, showing a good dose-effect relationship. The effect of acarbose on lactase was opposite to that of MAP-2; at 0.5 mg/mL, the activation rate of lactose by acarbose was slightly higher than that by MAP-2. However, with the increase in concentration, the activation rate not only decreased but also inhibited the activity of lactase when the concentration increased to 16 mg/mL, leading to lactose intolerance, and causing abdominal distension and abdominal pain in some diabetic patients. However, at present, there are few studies on lactase activity in vitro, especially the effect of polysaccharides on lactase activity is rarely reported. In previous research, it was found that the inhibition rate of MAP-2 on α-glucosidase was significantly higher than that of acarbose at the same concentration, and as an edible plant, Opuntia Milpa Alta is relatively safe [37]. At the same time, it also had a good activation effect on lactase. Therefore, in contrast, as an inhibitor of α-glucosidase, MAP-2 is not only more effective than acarbose, but also far less toxic and has fewer side effects than acarbose. It is an ideal type of α-glucosidase inhibitor for the future.

### 3.5. Determination of Hyaluronidase Activity

**Figure 8:**
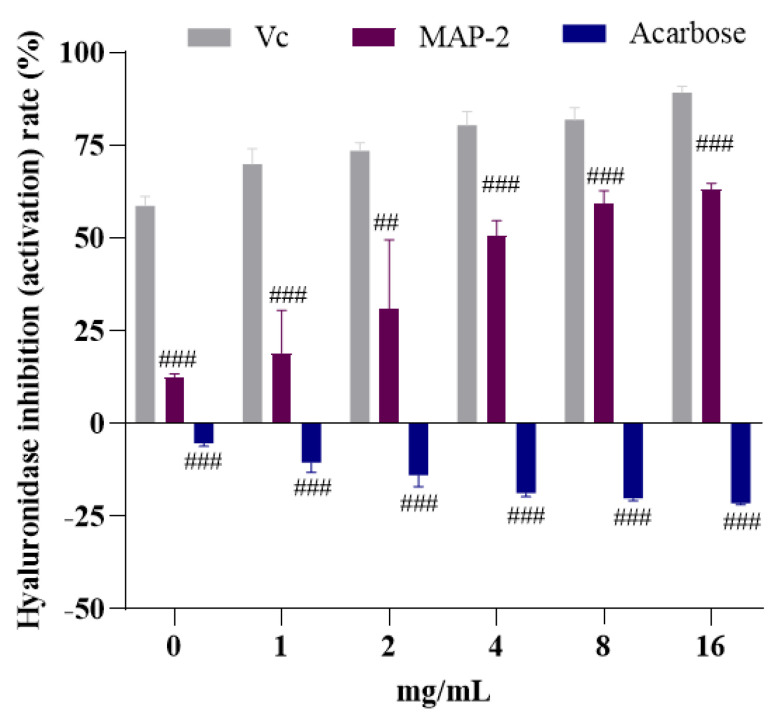
Effects of MAP−2 and Acarbose on hyaluronidase activity. ^##^ *p* < 0.01, ^###^ *p* < 0.001.

Hyaluronic acid is a polysaccharide with a large molecular weight, which mainly exists in the extracellular matrix, especially in soft connective tissue [38]. Hyaluronidase can randomly hydrolyze the β-1,4 bond between N-acetyl-D-glucosamine and D-glucuronic acid in transparent acid. It promotes cell proliferation, migration and may be involved in the progression of some malignant tumors [39]. Therefore, the inhibitory effect of hyaluronidase has been used as an in vitro test of anti-inflammatory and antiallergic activity. With acarbose widely used as an α-glucosidase inhibitor, a variety of toxic side effects have gradually emerged, in addition to common abdominal distension, diarrhea and abdominal pain [40], but also cause allergy and skin reactions [41]. To investigate whether MAP-2 could also produce this reaction, hyaluronidase was tested in vitro. The results showed that MAP-2 not only had a good inhibitory effect on hyaluronidase, but the inhibition rate increased with the increase in mass concentration (Figure 8). Compared with *Nostoc sphaericum* polysaccharides (IC_50_ 14.4 μg/mL) [42] and *Nostochopsis lobatus* polysaccharide (IC_50_ 7.18 μg/mL) [43], the IC_50_ of MAP-2 on hyaluronidase was higher. However, the inhibition rate of MAP-2 increased with the increase in concentration in the range of 0.5–16 mg/mL, the inhibition rate could reach 61.15% at 16 mg/mL, and the IC_50_ was 4.04 mg/mL. However, in the same mass concentration range, the effect of acarbose on hyaluronidase was opposite to that of MAP-2. With the increase in concentration, the activation rate increased gradually, indicating that the more acarbose input, the greater the probability of allergic reaction. Therefore, the combination of the lactase and hyaluronidase experiments found that MAP-2 is safer with non-toxic side effects, compared to acarbose, so may be more suitable for α-glucosidase inhibitor.

## 4. Conclusions

The consumption of cactus has been an admirable source of food nutrition and is widely used in medicine. In this study, based on a previous study, the process parameters for MAP-2 production were further optimized, and the optimum extraction conditions identified as: solid-liquid ratio 1:4, extraction temperature 90 °C, and extraction time 1 h. Under the optimal conditions, the yield of MAP-2 was 3.47 ± 0.062%, and when the concentration of MAP-2 was 16 mg/mL, the inhibition rate of α-glucosidase was 91 ± 0.623%, which was close to the predicted value. Through kinetic experiments, it was found that when the pH was 7.4 for 30 min, the inhibition rate of MAP-2 on α-glucosidase was the best, which was a reversible competitive inhibitory inhibition. In addition, in this study, in vitro model tests of lactase and hyaluronidase were established to confirm that MAP-2 can activate lactase activity and inhibit hyaluronidase. Compared with acarbose, MAP-2 not only inhibits α-glucosidase better, but also overcomes its adverse reactions. This conclusion provides strong theoretical and experimental evidence that MAP-2 can be used as an inhibitor of natural α-glucosidase.

## Figures and Tables

**Table 1:** Experimental design and results of the Box-Behnken design.

Std	Run	*X*_1_ (g/mL)	*X*_2_ (h)	*X*_3_ (°C)	Extraction Yield (%)	Inhibition α-Glucosidase Activity (%)
10	1	1:4	1.5	80	2.58	80.68
13	2	1:4	1.0	90	3.48	92.01
11	3	1:4	0.5	100	3.56	88.55
1	4	1:2	0.5	90	2.98	90.87
6	5	1:6	1.0	80	2.3	81.98
12	6	1:4	1.5	100	3.64	90.25
2	7	1:6	0.5	90	2.93	87.11
4	8	1:6	1.5	90	2.9	89.46
3	9	1:2	1.5	90	3.04	88.02
15	10	1:4	1.0	90	3.48	92.01
16	11	1:4	1.0	90	3.28	90.01
8	12	1:6	1.0	100	3.45	89.11
5	13	1:2	1.0	80	2.26	78.41
7	14	1:2	1.0	100	3.4	88.62
14	15	1:4	1.0	90	3.28	90.01
17	16	1:4	1.0	90	3.48	91.00
9	17	1:4	0.5	80	2.37	81.39

Note: *X*_1_, the solid-liquid ratio; *X*_2_, extraction time; *X*_3_, extraction temperature.

**Table 2:** Variance analysis of Box-Behnken design for MAP-2 yield.

Source	Sum of Squares	Df	Mean Square	F-Value	*p*-Value	
Model	3.38	9	0.3753	39.54	<0.0001 ***	significant
*X*_1_-Ratio of solid to liquid	0.0013	1	0.0013	0.1317	0.7274	
*X*_2_-Extraction time	0.0128	1	0.0128	1.35	0.2836	
*X*_3_-Extraction temperature	2.58	1	2.58	271.41	<0.0001 ***	
AB	0.0020	1	0.0020	0.2133	0.6582	
AC	0.0000	1	0.0000	0.0026	0.9605	
BC	0.0042	1	0.0042	0.4451	0.5261	
A^2^	0.4079	1	0.4079	42.97	0.0003 ***	
B^2^	0.0671	1	0.0671	7.07	0.0325 *	
C^2^	0.2350	1	0.2350	24.76	0.0016 **	
Residual	0.0664	7	0.0095			
Lack of Fit	0.0184	3	0.0061	0.5125	0.6952	not significant
R^2^	0.9807					
Adjusted R^2^	0.9559					
Pure Error	0.0480	4	0.0120			
Cor Total	3.44	16				

* Significant (*p* < 0.05); ** Very significant (*p* < 0.01); *** Extremely Significant (*p* < 0.001).

**Table 3:** Variance analysis of Box-Behnken design results of MAP-2 inhibiting α-glucosidase activity.

Source	Sum of Squares	Df	Mean Square	F-Value	*p*-Value	
Model	281.03	9	31.23	23.22	0.0002 ***	significant
*X*_1_-Ratio of solid to liquid	0.3785	1	0.3785	0.2815	0.6122	
*X*_2_-Extraction time	0.0300	1	0.0300	0.0223	0.8854	
*X*_3_-Extraction temperature	145.10	1	145.10	107.91	<0.0001 ***	
AB	6.76	1	6.76	5.03	0.0599	
AC	2.37	1	2.37	1.76	0.2258	
BC	1.45	1	1.45	1.08	0.3333	
A^2^	8.45	1	8.45	6.28	0.0406 *	
B^2^	2.24	1	2.24	1.66	0.2382	
C^2^	107.96	1	107.96	80.30	<0.0001 ***	
Residual	9.41	7	1.34			
Lack of Fit	5.41	3	1.80	1.80	0.2860	not significant
R^2^	0.9676					
Adjusted R^2^	0.9259					
Pure Error	4.00	4	1.0000			
Cor Total	290.44	16				

* Significant (*p* < 0.05); *** Extremely significant (*p* < 0.001).

**Table 4:** Predicted and experimental values of the responses at optimum and modified conditions.

	*X*_1_ (g/mL)	*X*_2_ (h)	*X*_3_ (°C)	Yield (%)	Inhibition α-Glucosidase Activity (%)
Optimum conditions	1:4	1.1	95	3.64	91.87 (predicted)
Modified conditions	1:4	1.0	90	3.47	91.13 ± 0.623 (actual)

*X*_1_: ratio of solid to liquid, *X*_2_: extraction time, *X*_3_: extraction temperature.

## Data Availability

Data is contained within this article.

## References

[1] Li C., Hung Y.-J., Qamruddin K., Aziz M.F.A., Stein H., Schmidt B. (2011). International Noninterventional Study of Acarbose Treatment in Patients with Type 2 Diabetes Mellitus. Diabetes Res. Clin. Pract..

[2] Puls W., Keup U., Krause H.P., Thomas G., Hoffmeister F. (1977). Glucosidase Inhibition. A New Approach to the Treatment of Diabetes, Obesity, and Hyperlipoproteinaemia. Naturwissenschaften.

[3] Zhu J., Chen C., Zhang B., Huang Q. (2020). The Inhibitory Effects of Flavonoids on α-Amylase and α-Glucosidase. Crit. Rev. Food Sci. Nutr..

[4] Wu J., Shi S., Wang H., Wang S. (2016). Mechanisms Underlying the Effect of Polysaccharides in the Treatment of Type 2 Diabetes: A Review. Carbohydr. Polym..

[5] Uzui H., Nakano A., Mitsuke Y., Geshi T., Sakata J., Sarazawa K., Morishita T., Satou T., Ishida K., Lee J.-D. (2011). Acarbose Treatments Improve Arterial Stiffness in Patients with Type 2 Diabetes Mellitus. J. Diabetes Investig..

[6] Kasthuri S., Poongothai S., Anjana R.M., Selvakumar J., Muthukumar S., Kayalvizhi S., Tariq S., Honey E., Gupta P.K., Venkatesan U. (2021). Comparison of Glycemic Excursion Using Flash Continuous Glucose Monitoring in Patients with Type 2 Diabetes Mellitus Before and After Treatment with Voglibose. Diabetes Technol. Ther..

[7] Scott L.J., Spencer C.M. (2000). Miglitol: A Review of Its Therapeutic Potential in Type 2 Diabetes Mellitus. Drugs.

[8] Fujisawa T., Ikegami H., Inoue K., Kawabata Y., Ogihara T. (2005). Effect of Two Alpha-Glucosidase Inhibitors, Voglibose and Acarbose, on Postprandial Hyperglycemia Correlates with Subjective Abdominal Symptoms. Metabolism..

[9] Shu C., Ge H., Song M., Chen J., Zhou H., Qi Q., Wang F., Ma X., Yang X., Zhang G. (2014). Discovery of Imigliptin, a Novel Selective DPP-4 Inhibitor for the Treatment of Type 2 Diabetes. ACS Med. Chem. Lett..

[10] Dash R.P., Babu R.J., Srinivas N.R. (2018). Reappraisal and Perspectives of Clinical Drug-Drug Interaction Potential of α-Glucosidase Inhibitors Such as Acarbose, Voglibose and Miglitol in the Treatment of Type 2 Diabetes Mellitus. Xenobiotica Fate Foreign Compd. Biol. Syst..

[11] Uchida T., Kawai J., Fujitani Y., Kawamori R., Watada H., Hirose T. (2010). Efficacy and Adverse Effects of Low-Dose Nateglinide in Early Type 2 Diabetes: Comparison with Acarbose in a Crossover Study. Diabetol. Int..

[12] Zhang M., Yang R., Yu S., Zhao W. (2022). A Novel A-glucosidase Inhibitor Polysaccharide from *Sargassum Fusiforme*. Int. J. Food Sci. Technol..

[13] Fu X., Yang H., Ma C., Li X., Li D., Yang Y., Xu Y., Wang L. (2020). Characterization and Inhibitory Activities on α-Amylase and α-Glucosidase of the Polysaccharide from Blue Honeysuckle Berries. Int. J. Biol. Macromol..

[14] Zhang Z., Kong F., Ni H., Mo Z., Wan J.-B., Hua D., Yan C. (2016). Structural Characterization, α-Glucosidase Inhibitory and DPPH Scavenging Activities of Polysaccharides from Guava. Carbohydr. Polym..

[15] Guo Q., Chen Z., Santhanam R.K., Xu L., Gao X., Ma Q., Xue Z., Chen H. (2019). Hypoglycemic Effects of Polysaccharides from Corn Silk (Maydis Stigma) and Their Beneficial Roles via Regulating the PI3K/Akt Signaling Pathway in L6 Skeletal Muscle Myotubes. Int. J. Biol. Macromol..

[16] Chen J., Zhang X., Huo D., Cao C., Li Y., Liang Y., Li B., Li L. (2019). Preliminary Characterization, Antioxidant and α-Glucosidase Inhibitory Activities of Polysaccharides from Mallotus Furetianus. Carbohydr. Polym..

[17] Wu L., Sun H., Hao Y., Zheng X., Song Q., Dai S., Zhu Z. (2020). Chemical Structure and Inhibition on α-Glucosidase of the Polysaccharides from Cordyceps Militaris with Different Developmental Stages. Int. J. Biol. Macromol..

[18] A Study on the Time-Effect and Dose-Effect Relationships of Polysaccharide from Opuntia Dillenii against Cadmium-Induced Liver Injury in Mice—PubMed. https://pubmed.ncbi.nlm.nih.gov/35564063/.

[19] Multisociety Consensus Quality Improvement Revised Consensus Statement for Endovascular Therapy of Acute Ischemic Stroke—PubMed. https://pubmed.ncbi.nlm.nih.gov/29786478/.

[20] Zhang H., Wu Q., Qin X. (2019). Camellia Nitidissima Chi Flower Extracts Inhibit α-Amylase and α-Glucosidase: In Vitro by Analysis of Optimization of Addition Methods, Inhibitory Kinetics and Mechanisms. Process Biochem..

[21] Kumar R., Henrissat B., Coutinho P.M. (2019). Intrinsic Dynamic Behavior of Enzyme:Substrate Complexes Govern the Catalytic Action of β-Galactosidases across Clan GH-A. Sci. Rep..

[22] Möller S., Schmidtke M., Weiss D., Schiller J., Pawlik K., Wutzler P., Schnabelrauch M. (2012). Synthesis and Antiherpetic Activity of Carboxymethylated and Sulfated Hyaluronan Derivatives. Carbohydr. Polym..

[23] Xu Q., Shen Y., Wang H., Zhang N., Xu S., Zhang L. (2013). Application of Response Surface Methodology to Optimise Extraction of Flavonoids from Fructus Sophorae. Food Chem..

[24] Ye C.-L., Jiang C.-J. (2011). Optimization of Extraction Process of Crude Polysaccharides from Plantago Asiatica L. by Response Surface Methodology. Carbohydr. Polym..

[25] Cui F.-J., Qian L.-S., Sun W.-J., Zhang J.-S., Yang Y., Li N., Zhuang H.-N., Wu D. (2018). Ultrasound-Assisted Extraction of Polysaccharides from Volvariella Volvacea: Process Optimization and Structural Characterization. Molecules.

[26] Muralidhar R.V., Chirumamila R.R., Marchant R., Nigam P. (2001). A Response Surface Approach for the Comparison of Lipase Production by Candida Cylindracea Using Two Different Carbon Sources. Biochem. Eng. J..

[27] Martínez-Felipe A., Cook A.G., Wallage M.J., Imrie C.T. (2014). Hydrogen Bonding and Liquid Crystallinity of Low Molar Mass and Polymeric Mesogens Containing Benzoic Acids: A Variable Temperature Fourier Transform Infrared Spectroscopic Study. Phase Transit..

[28] Hu J., Liu Y., Cheng L., Shi R., Qayum A., Bilawal A., Gantumur M.-A., Hussain M.A., Jiang Z., Tian B. (2020). Comparison in Bioactivity and Characteristics of Ginkgo Biloba Seed Polysaccharides from Four Extract Pathways. Int. J. Biol. Macromol..

[29] Li F., Wei Y., Liang L., Huang L., Yu G., Li Q. (2021). A Novel Low-Molecular-Mass Pumpkin Polysaccharide: Structural Characterization, Antioxidant Activity, and Hypoglycemic Potential. Carbohydr. Polym..

[30] Hsu W., Hsu T., Lin F., Cheng Y., Yang J.P. (2013). Separation, Purification, and α-Glucosidase Inhibition of Polysaccharides from Coriolus Versicolor LH1 Mycelia. Carbohydr. Polym..

[31] Zhang L.-L., Han L., Yang S.-Y., Meng X.-M., Ma W.-F., Wang M. (2019). The Mechanism of Interactions between Flavan-3-Ols against a-Glucosidase and Their in Vivo Antihyperglycemic Effects. Bioorganic Chem..

[32] He M., Zhai Y., Zhang Y., Xu S., Yu S., Wei Y., Xiao H., Song Y. (2022). Inhibition of α-Glucosidase by Trilobatin and Its Mechanism: Kinetics, Interaction Mechanism and Molecular Docking. Food Funct..

[33] Stourman N., Moore J. (2018). Analysis of Lactase in Lactose Intolerance Supplements. Biochem. Mol. Biol. Educ. Bimon. Publ. Int. Union Biochem. Mol. Biol..

[34] Bayless T.M., Brown E., Paige D.M. (2017). Lactase Non-Persistence and Lactose Intolerance. Curr. Gastroenterol. Rep..

[35] Voisin M.R., Borici-Mazi R. (2016). Anaphylaxis to Supplemental Oral Lactase Enzyme. Allergy Asthma Clin. Immunol..

[36] Singla R.K., Singh R., Dubey A.K. (2016). Important Aspects of Post-Prandial Antidiabetic Drug, Acarbose. Curr. Top. Med. Chem..

[37] Chen Y., Zhao B., Huang X., Zhan J., Zhao Y., Zhou M., Guo L. (2011). Purification and Neuroprotective Effects of Polysaccharides from Opuntia Milpa Alta in Cultured Cortical Neurons. Int. J. Biol. Macromol..

[38] Weigel P.H., Hascall V.C., Tammi M. (1997). Hyaluronan Synthases. J. Biol. Chem..

[39] Noble P.W., Lake F.R., Henson P.M., Riches D.W. (1993). Hyaluronate Activation of CD44 Induces Insulin-like Growth Factor-1 Expression by a Tumor Necrosis Factor-Alpha-Dependent Mechanism in Murine Macrophages. J. Clin. Investig..

[40] Hollander P. (1992). Safety Profile of Acarbose, an Alpha-Glucosidase Inhibitor. Drugs.

[41] Kono T., Hayami M., Kobayashi H., Ishii M., Taniguchi S. (1999). Acarbose-Induced Generalised Erythema Multiforme. Lancet Lond. Eng..

[42] Yamaguchi Y., Koketsu M. (2016). Isolation and Analysis of Polysaccharide Showing High Hyaluronidase Inhibitory Activity in Nostochopsis Lobatus MAC0804NAN. J. Biosci. Bioeng..

[43] Yamaguchi Y., Sakamoto T., Koketsu M. (2015). Comparison of Anti-Hyaluronidase Activities and Sugar Compositions of Extracts from Four Edible Species of *Nostoc* (Cyanobacteria). Algal Resour..

